# Genome wide association study discovers genomic regions involved in resistance to soybean cyst nematode (*Heterodera glycines*) in common bean

**DOI:** 10.1371/journal.pone.0212140

**Published:** 2019-02-07

**Authors:** Shalu Jain, Susilo Poromarto, Juan M. Osorno, Phillip E. McClean, Berlin D. Nelson

**Affiliations:** 1 Department of Plant Pathology, North Dakota State University, Fargo, North Dakota, United States of America; 2 Department of Agrotechnology, Sebelas Maret University, Surakarta, Jawa Tengah, Indonesia; 3 Department of Plant Sciences, North Dakota State University, Fargo, North Dakota, United States of America; Università Politecnica delle Marche, ITALY

## Abstract

Common bean (*Phaseolus vulgaris* L.) is an important high protein crop grown worldwide. North Dakota and Minnesota are the largest producers of common beans in the USA, but crop production is threatened by soybean cyst nematode (SCN; *Heterodera glycines* Ichinohe) because most current cultivars are susceptible. Greenhouse screening data using SCN HG type 0 from 317 plant introductions (PI’s) from the USDA core collection was used to conduct a genome wide association study (GWAS). These lines were divided into two subpopulations based on principal component analysis (Middle American vs. Andean). Phenotypic results based on the female index showed that accessions could be classified as highly resistant (21% and 27%), moderately resistant (51% and 48%), moderately susceptible (27% and 22%) and highly susceptible (1% and 3%) for Middle American and Andean gene pools, respectively. Mixed models with two principal components (PCs) and kinship matrix for Middle American genotypes and Andean genotypes were used in the GWAS analysis using 3,985 and 4,811 single nucleotide polymorphic (SNP) markers, respectively which were evenly distributed across all 11 chromosomes. Significant peaks on Pv07, and Pv11 in Middle American and on Pv07, Pv08, Pv09 and Pv11 in Andean group were found to be associated with SCN resistance. Homologs of soybean *rhg1*, a locus which confers resistance to SCN in soybean, were identified on chromosomes Pv01 and Pv08 in the Middle American and Andean gene pools, respectively. These genomic regions may be the key to develop SCN-resistant common bean cultivars.

## Introduction

Common bean (*Phaseolus vulgaris* L.) is one of the most important legume crops worldwide as a significant source of protein, iron, fiber and other micronutrients in human diets [1, 2]. The United States is the fourth-largest producer of dry edible beans and contributes ~6% of total world output (https://www.nass.usda.gov/), which includes both the *Phaseolus* beans and species belonging to *Vigna* genera [3]. North Dakota is the largest producer of common bean in the United States with 32% of the total production. Common bean is susceptible to different pests and pathogens and recently, production in North Dakota was challenged by soybean cyst nematode (SCN) (*Heterodera glycines* Ichinohe), a new bean pathogen first identified on soybean in North Dakota in 2003 [4].

SCN is considered the most devastating soybean [*Glycine max* (L.) Merr.] pest and causes severe seed yield losses worldwide [5]. SCN was found in counties in North Dakota and Minnesota where common bean and soybeans are grown in close proximity. This provides potential for further spread of the pathogen to common bean fields [6]. SCN infested soil can be disseminated by farm machinery, vehicles, wind, water, animals and farm workers. With further spread of SCN through the production region, the pathogen could seriously threaten common bean production in the region. As observed in soybean, there are often no obvious above ground symptoms of SCN infection, yet seed yield can be reduced. When above ground symptoms are visible such as patches of yellow and stunted plants, the damage is usually much greater. In common bean, field research has demonstrated that SCN can reduce seed yield up to 50% in susceptible pinto, navy and kidney bean cultivars [7, 8]. Environmental conditions and soil egg levels are important factors in seed yield losses. There are different virulent types of SCN in this major production area, but at present, SCN HG Type 0 is the most common [9]. However, HG Type 2, which is virulent against the SCN resistance gene commonly used in soybean is starting to appear in fields [9].

In soybean, the use of SCN resistant cultivars is the principal management strategy and a similar strategy may be needed for common bean. The development and use of resistant cultivars could be an efficient and environmentally friendly method of managing SCN if sources of genetic resistance are available. Most of the soybean cultivars grown in USA use resistance derived primarily from plant introduction PI 88788 which requires the *rhg1* gene for managing SCN [10]. However, continuous use of a single source of resistance generally drives the SCN population to evolve in order to overcome host resistance. In soybean, SCN resistance is largely based on the identification of two QTLs, *rhg1* on chromosome 18 [11], and *Rhg4* on chromosome 8 [12]. Cloning of the *rhg1* locus indicated that a genomic region containing copy number variation (CNV) of three tandem genes, an amino acid transporter, an alpha- soluble Nethylmaleimide-sensitive factor (NSF)-Attachment Protein (SNAP), and a gamma bisabolene synthase 1-related protein confers resistance to SCN in accessions such as PI88788 [11]. Peking-type resistance requires an additional locus, *Rhg4*, and a new allele of α-SNAP [13, 14]. A cytosolic enzyme, serine hydroxyl methyl transferase (SHMT) is encoded by *Rhg4* locus [12]. Other novel QTLs on soybean chromosome 10 (qSCN10) and chromosome 11 (qSCN11) were also identified [15, 16, 17].

The common bean USDA core collection represents an invaluable source of genetic diversity that can be utilized to find PI’s with resistance to SCN. This collection represents plant introduction accessions from two gene pools originating from Central and South America [18]. Few studies have explored the phenotypic variation associated with SCN resistance in common bean [7, 19]. Smith and Young [20] conducted a greenhouse study to evaluate 20 common bean genotypes for SCN resistance and demonstrated that some Mesoamerican genotypes (Dorado, Burke, Porrillo Sintetico and Chase) were more tolerant than Andean genotypes (G122, G19833, Contender, Taylor Horticultural, and Tendercrop) and other Mesoamerican genotypes (Maverick, Matterhorn, Cornell 2114–12, and Tio Canela 75), based on cyst numbers on the roots. Development of SCN resistant breeding lines/cultivars is a long process requiring considerable screening with the nematodes. Recent developments in molecular marker techniques can help to shorten the timeline of cultivar development. A key step in developing SCN-resistant cultivars is elucidation of the genetic basis of resistance.

The genome-wide association study (GWAS) strategy is considered an efficient approach for understanding the genetics behind a complex trait such as SCN resistance [21]. Through association mapping, two significant loci corresponding to the soybean *rhg1* and *FGAM1* genes were detected on chromosome 18 along with a third locus at the opposite end of chromosome 18 [21]. The GWAS approach has been applied to verify previously identified SCN resistance QTL in cultivated soybean populations and novel candidate genes were identified [22, 23]. GWAS can offer higher resolution mapping and uncover a broader range of genetic recombination events that occur over time; however, its ability to map rare QTL alleles is lower than recombinant inbred populations [24, 25]. Due to the broad genetic diversity and the potential source of new allelic variation, the USDA common bean core collection is an ideal candidate for association mapping based studies [18].

Recently, Wen [26] conducted GWAS to detect SNPs associated with SCN resistance in the common bean core collection using HG types 2.5.7 and 1.3.3.5.6.7. Wen [26] reported SNPs on chromosome 1 significantly associated with resistance to HG 2.5.7 and a novel signal on chromosome 7 associated with resistance to the HG type 1.2.3.5.6.7. To date, the genetic basis of resistance/susceptibility to SCN HG type 0 has not been reported for either cultivated populations of common bean or wild relatives of bean. In this study, a genome wide association analysis was conducted on a set of 317 *P*. *vulgaris* PI accessions from the USDA common bean core collection with the aims of (1) identifying genomic regions involved in SCN HG Type 0 resistance and (2) identifying molecular markers and candidate genes significantly associated with SCN HG Type 0 resistance.

## Materials and methods

### Plant material and phenotyping

The common bean USDA core collection consisting of 423 PI’s was obtained from the USDA Western Regional Plant Introduction Station in Pullman, WA. The core collection consists of mostly land races and ten wild accessions. The accessions in core collection were collected from Mexico (200) and Central and South America (223). It consists of two distinct gene pools namely the Middle American and Andean gene pool. The population of SCN HG type 0 was obtained from naturally infested soil collected from a soybean field in Richland Co., North Dakota [7] and maintained on Barnes (highly susceptible soybean variety) in greenhouse. Screening for resistance to SCN HG type 0 was conducted in greenhouse using the method described by Poromarto and Nelson [7]. Briefly, seeds were surface disinfected with 1.0% NaOCl for one minute, rinsed with water then germinated on seed germination paper for 3 days. Healthy seedlings were transplanted into a 1 x 2.5 cm hole in autoclaved sandy soil (La Prairie silt loam) in plastic “Cone-tainers” Type SC10 Super Cell (Stuewe & Sons, Inc., Corvallis, OR, USA). There was one seedling per container and seedlings were immediately inoculated with SCN HG 0 by placing 2,000 freshly harvested eggs in a water solution directly around the seedling then the seedlings were covered with soil. Cone-tainers” were placed in autoclaved sand in 30.5 cm dia x 30.5 cm depth plastic pots (Cambro, Huntington Beach, CA) immersed in a water bath at 27 ± 2° C in the greenhouse. Because of limited space in the water bath, testing of PI’s was completed at different times with approximately 50 PI’s in an individual test. The experimental design was a randomized complete block with blocks being the two plastic pots. There were 4 replications of each PI in each test. There were two plants of Barnes in each pot as the susceptible soybean check. Barnes is equal in susceptibility to Lee74, which is commonly used as a susceptible check in screening soybean for SCN resistance [7]. Accessions showing inconsistent results were repeated one more time.

Plants were grown for 30 days under natural and artificial light with high pressure sodium lamps (1,000 μE. m-2.s-1) for 16 h/day. SCN females were harvested from the roots of individual plants as described by Poromarto and Nelson [7] and counted. For each individual experiment, a female index (FI) was calculated for each accession [27].

### Genome wide association study (GWAS)

A total of 5398 BARCBean6K SNPs data [28] was used for the GWAS analysis. A total of 317 accessions were used in this study based on availability of both genotypic and phenotypic data and further divided into two subpopulations based on principal component analysis (PCA) due to previous knowledge of two gene pools in this collection [18, 29]. Population structure, kinship matrix and genome wide association mapping was conducted separately on each group in JMP Genomics 8.1 (SAS 2015). Markers having minor allele frequency <0.01 and > 50% missing genotypic data were removed from the final analysis. The population structure which represents the genetic similarity among genotypes was computed using principal component analysis (PCA). A kinship (K) matrix that represents the proportion of shared alleles for all pairwise comparisons in each population was computed. Six regression models were generated for each subpopulation: (1) naive model, (2) kinship (K), (3) principal component (PC) 1, (4) PC1 and K, (5) PC1 and PC2, and (6) PC1, PC2 and K. The best model was determined according to lowest Bayesian Information Criterion (BIC) [30, 31]. Marker-trait associations were considered significant at P ≤ 0.001. By assuming the identified genomic regions acted additively, a forward stepwise linear regression model with the FI estimates as dependent variables and the significant SNP markers as explanatory variables was constructed in JMP Genomics 8.1 (SAS 2015). Adjusted *R*^2^ values were estimated from the linear regression model representing the percentage of phenotypic variation explained by the associated SNPs. Linkage disequilibrium for all pairwise comparisons between intrachromosomal SNPs was computed as the squared correlation coefficient for each of the marker pairs using the maximum likelihood algorithm with JMP Genomics 8.1 (SAS 2015).

### Candidate gene prediction and sequence analysis

Candidate genes were selected based on two criteria: (i) a homology based search for *priori* SCN resistance genes in soybean close to markers identified in common bean and (ii) genes located within 100-Kb window regions of significant SNPs. To identify macro- and micro-synteny between soybean SCN-related QTL (*rhg1*) and bean regions in this experiment, the protein sequences of the *rhg1* in soybean (http://soybase.org) were blasted against the common bean reference version 2.1 genome (https://phytozome.jgi.doe.gov). Significant hits (E-values less than 1e^-40^) were considered as homologous genes. Genomic sequences of *P*. *vulgaris* and *G*. *max* were downloaded from Phytozome and comparative mapping analysis was conducted over a 1.43 Mb region using AutoGRAPH [32]. The protein sequences encoded by the predicated SNAP genes were retrieved from the Phytozome database (https://phytozome.jgi.doe.gov). SNAP sequences obtained from common bean were also analyzed using the NCBI Conserved Domain Database for tetratricopeptide repeat (TPR) domains (http://www.ncbi.nlm.nih.gov/Structure/cdd/cdd.shtml). Functional annotation of the genes was conducted using SMARTBLAST (https://blast.ncbi.nlm.nih.gov/smartblast/smartBlast.cgi) and multiple sequence alignments were conducted with Clustal Omega [33].

## Results and discussion

### Sources of SCN HG Type 0 resistance

**Fig 1:**
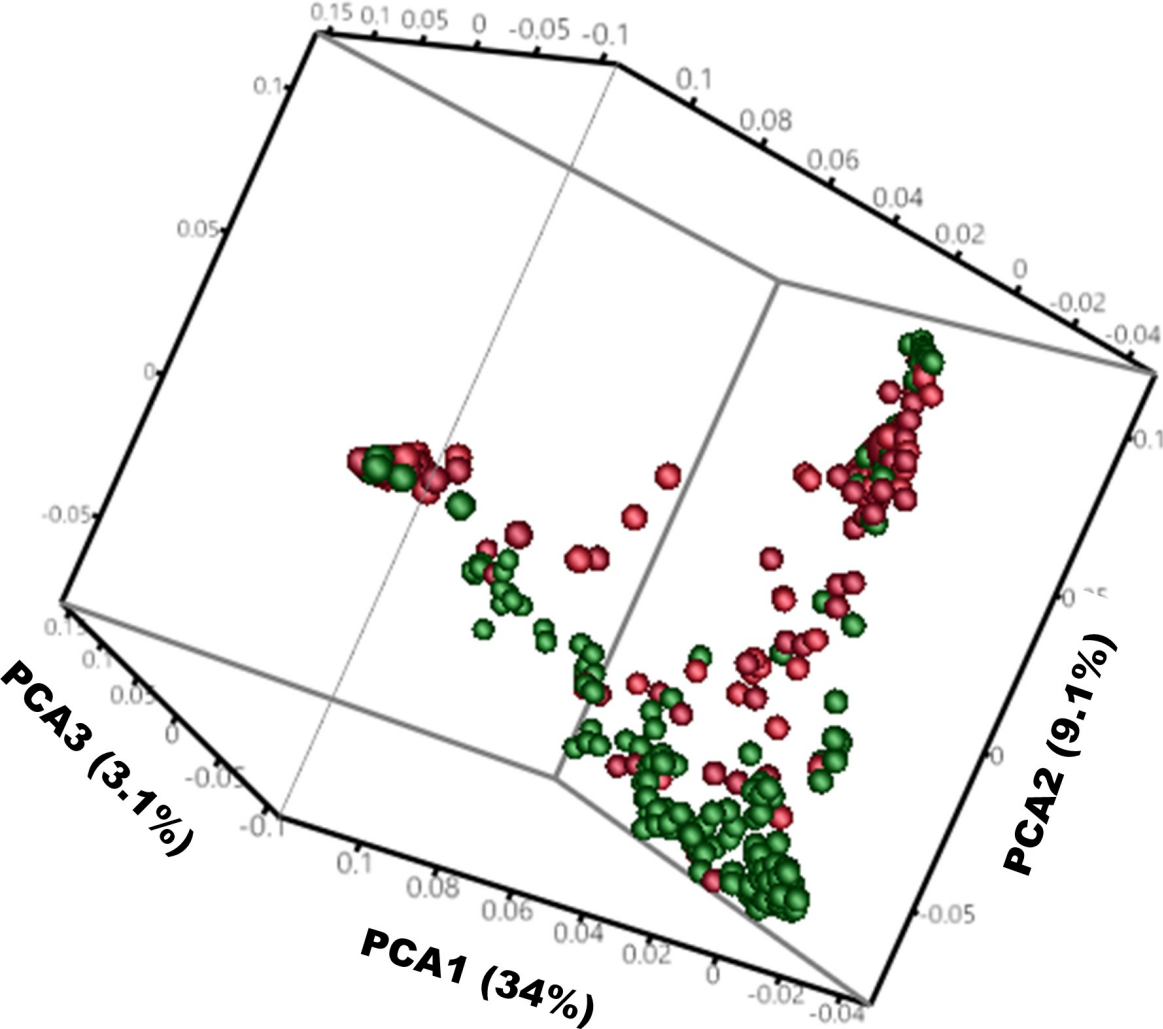
Principal component analysis (PCA) obtained from 5,061 polymorphic SNPs, indicating the population structure in 317 common bean PI accessions. PCA1, PCA2 and PCA3 explained 34%, 9.1%, and 3.1% of the variation, respectively. The red colored pixels represent accessions collected from Central-South America and green colored pixels represent accessions collected from Mexico, however, accessions grouped together based on genepools present in the core collection.

**Fig 2:**
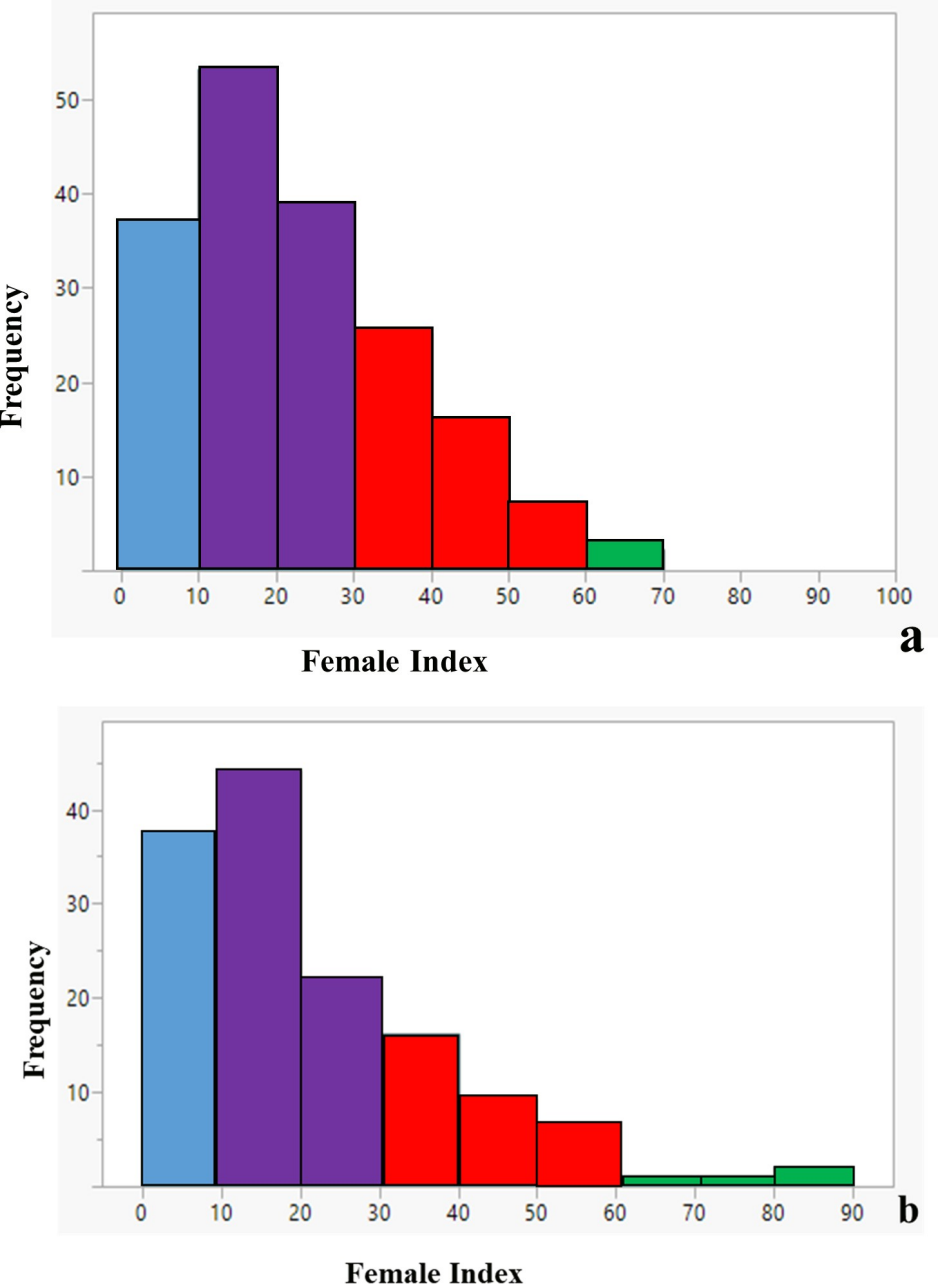
Distribution of the soybean cyst nematode female index (FI). **a.** Middle American subpopulation of common bean consisting 179 PI accessions and **b.** Andean subpopulation consisting of 138 PI accessions. Resistant (FI < 10; blue), moderately resistant (FI 10 to 30; purple), moderately susceptible (FI 31 to 60; red), susceptible (FI > 60; green).

Phenotypic data for 317 accessions was used in this study, and significant variation in SCN resistance was observed in the common bean core collection with FI’s ranging from 3 to 104 with a mean FI of 22.4. Due to the presence of two strongly distinct gene pools, the Middle American and Andean, these accessions were separated into two groups based on principal component analysis as suggested by Mamidi et al. [29] using SNP data. First principal component (PC1) explained 34% of the variation and PC2 explained 9.1% of the variation (Fig 1). A Middle American group consisted of 179 accessions mostly originating from Mexico and few lines from Central and South America. An Andean group consisted of 138 accessions mostly originating from Central and South America and some from Mexico. McClean et al. [18] obtained similar results in population structure using 171 accessions from the core collection. Phenotypic results indicated that a large number of accessions were resistant to SCN. Out of total accessions, 21% and 27% were classified as highly resistant (FI < 10), 51% and 48% were classified as moderately resistant (FI 10 to 30), 27% and 22% were classified as moderately susceptible (FI 31 to 60) and 1% and 3% were classified as highly susceptible (FI > 60) for Middle American and Andean subpopulations, respectively based on the FI resistance scale of Schmitt and Shannon [34] developed for soybean (Fig 2A and 2B). A resistance scale for common bean has not been developed, but the Schmitt and Shannon scale can be applied in bean for the purpose of identifying and selecting genotypes that allow limited SCN reproduction on the roots, a key factor to prevent seed yield loss. Since the distribution of FI values was continuous, the most likely genetic basis of resistance in common bean is quantitative in nature. Likewise, many studies conducted on soybean also indicated a complex genetic architecture of SCN resistance [35, 36]. Many of the highly resistant accessions in the common bean core collection could be used in breeding programs to start introgression of resistance into elite/adapted germplasm and commercial cultivars.

### Genome wide association analysis

**Fig 3:**
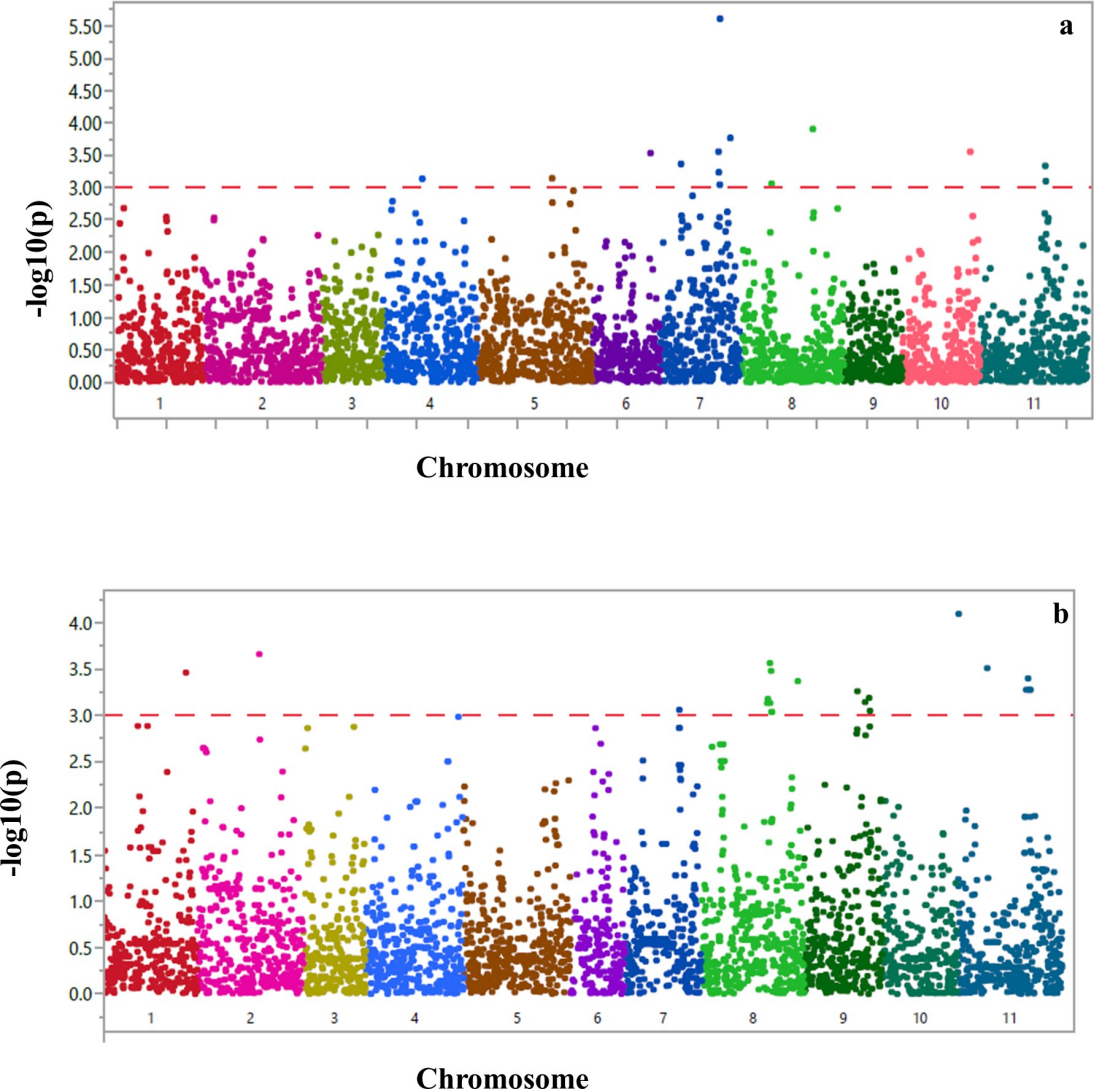
Genome wide association mapping analysis of disease reaction to soybean cyst nematode HG type 0. **(a)** Middle American and **(b)** Andean subpopulations of the common bean core collection. *Phaseolus vulgaris* chromosomes are listed on the x-axis. A -log10(p) scale of significance is represented on the y-axis with the red horizontal line representing the significance threshold of -log_10_(p) = 3. The colored pixels represent individual SNP markers.

A total of 3,985 and 4,811 high quality SNP markers were selected for Middle American and Andean subpopulations, respectively for GWAS. SNPs were evenly distributed on all eleven chromosomes. Accounting for the population structure and familial relationship between individuals in the GWAS was conducted to reduce the number of false-positive associations. Therefore, it was critical to select the proper number of PCs and dimensions for the GWAS. Population structure was inferred using principal component analysis (PCA). The PCA showed that PCA1, PCA2 and PCA3 explained 13%, 6.5% and 5.2% of the genotype variation, respectively for Middle American group while the PCA analysis for Andean group showed that PC1, PC2 and PC3 resolved 49.4%, 5.2% and 2.7% of the variation, respectively. The familial relatedness between the accessions was estimated using an identity-by-state (K matrix). Based on the lowest BIC values of regression models, including two PCs with kinship (PC1, PC2 plus K) was the best fit for Middle American and Andean subpopulations to conduct GWAS (Table 1). The most significant peaks were observed on common bean chromosomes Pv07 and Pv11 for the Middle American subpopulation and on Pv07, Pv08, Pv09 and Pv11 for the Andean subpopulation suggesting a unique or quantitative genetic control of SCN resistance in the two subpopulations (Fig 3A and 3B). In addition to two significant peaks, six SNPs (Table 2) closely associated with SCN resistance on chromosome Pv04, Pv05, Pv06, Pv08 and Pv10 were identified for the Middle American subpopulation. Similarly, two additional SNPs (Table 3) were closely associated with SCN resistance on chromosome Pv01 and Pv02 were identified for the Andean subpopulation. The -log_10_(p) of the significant markers ranged from 3.05–5.6 and explained between 9.2–17.6% of the phenotypic variation (R^2^) per marker for Middle American subpopulation (Table 2). The -log10(p) of the significant markers ranged from 3.04–4.1 and explained between 8.1–11.2% of the phenotypic variation (R^2^) per marker for the Andean subpopulation (Table 3). The most significant peak on chromosome 8 has 9 SNPs that are located within 3.4 Mb from each other and were found to be in linkage disequilibrium with squared correlation coefficient values of 0.92–1 (S1 Fig). It also suggests there are different resistance loci except for three common loci on Pv07, Pv08 and Pv11 in these two gene pools.

**Table 1:** Bayesian information criterion (BIC) for tested association models in the genome association analysis. The model with lowest BIC value was selected.

Model	BIC values
**Middle American**	
Naive*	1467.12
K	1447.61
PC1	1459.16
PC1 & K	1443.60
PC1 & PC2	1461.23
PC1, PC2 & K	1440.48
**Andean**	
Naive*	1177.63
K	1167.78
PC1	1182.46
PC1 & K	1167.68
PC1 & PC2	1187.38
PC1, PC2 & K	1167.67

*Naïve- No kinship (K) or principal component (PC) accounted in the analysis

**Table 2:** Significant markers associated with a resistant reaction to SCN HG type 0 in the Middle American subpopulation of the common bean core collection.

SNP	Chr*	Major Allele	Minor Allele	MAF**	-Log10(p)	R^2^
sc00222ln388011_3381_C_T_149591563	4	T	C	0.23	3.14	0.094
sc00505ln218075_188822_C_A_231896501	5	A	C	0.16	3.15	0.119
sc00012ln1449677_470064_T_C_20535929	6	C	T	0.02	3.54	0.108
sc00069ln687552_417242_G_A_70966666	7	A	G	0.42	5.61	0.176
sc00028ln943921_691222_A_G_39017987	7	G	A	0.18	3.77	0.115
sc00069ln687552_531119_G_T_71080543	7	T	G	0.36	3.56	0.108
sc00555ln205282_74219_C_T_242362884	7	T	C	0.41	3.37	0.128
sc00069ln687552_523214_A_G_71072638	7	G	A	0.41	3.24	0.097
sc00069ln687552_445042_A_G_70994466	7	G	A	0.46	3.05	0.092
sc00944ln124871_116143_A_G_304183827	8	G	A	0.13	3.91	0.122
sc00466ln235663_107380_G_A_222922045	8	A	G	0.08	3.06	0.092
sc00309ln320131_4769_C_T_179942914	10	T	C	0.22	3.56	0.109
sc00331ln303842_258016_G_A_187046890	11	A	G	0.36	3.34	0.108
sc00331ln303842_243301_T_C_187032175	11	C	T	0.35	3.10	0.093

*Chromosome

**Marker allele frequency

**Table 3:** Significant markers associated with a resistant reaction to SCN HG type 0 in the Andean subpopulation of the common bean core collection.

SNP	Chr*	Major Allele	Minor Allele	MAF**	-Log10(p)	R^2^
sc00022ln1003704_109623_C_T_32611495	1	T	C	0.27	3.46	0.11
sc00265ln348200_251662_T_C_165499786	2	C	T	0.46	3.66	0.12
sc00394ln266395_223173_T_C_204976825	7	C	T	0.13	3.06	0.08
sc00615ln187153_14670_A_G_254141142	8	G	A	0.24	3.56	0.10
sc00874ln135217_24012_A_G_294993727	8	G	A	0.23	3.56	0.09
sc00615ln187153_48449_C_A_254174921	8	A	C	0.23	3.56	0.09
sc00915ln129006_11903_A_C_300404206	8	C	A	0.22	3.48	0.09
sc00091ln623366_105511_A_G_85246735	8	G	A	0.15	3.37	0.09
sc03015ln25795_24227_A_G_424454518	8	G	A	0.20	3.18	0.08
sc00303ln322736_322161_G_A_178330165	8	A	G	0.20	3.13	0.08
sc01886ln53823_3577_A_G_381672594	8	G	A	0.20	3.13	0.08
sc00977ln121615_117414_C_A_308244782	8	A	C	0.23	3.04	0.10
sc01150ln100434_26250_T_G_327275921	8	G	T	0.23	3.04	0.10
sc00074ln679062_300164_A_G_74264487	9	G	A	0.30	3.26	0.09
sc00011ln1496550_816617_T_G_19385932	9	G	T	0.26	3.19	0.08
sc00011ln1496550_257154_A_G_18826469	9	G	A	0.26	3.14	0.08
sc00011ln1496550_1068436_G_A_19637751	9	A	G	0.13	3.05	0.08
sc01428ln78433_61756_G_A_352114770	11	A	G	0.39	4.10	0.11
sc04083ln13522_9316_G_A_444539981	11	A	G	0.18	3.51	0.10
sc00331ln303842_149404_C_A_186938278	11	A	C	0.42	3.40	0.09
sc00331ln303842_263624_C_A_187052498	11	A	C	0.40	3.28	0.09
sc00331ln303842_45835_T_G_186834709	11	G	T	0.40	3.28	0.09
sc01867ln54851_44400_C_T_380677834	11	T	C	0.40	3.28	0.09

*Chromosome

**Marker allele frequency

Seven significant markers (sc00331ln303842_258016_G_A_187046890, sc00309ln320131_4769_C_T_179942914, sc00944ln124871_116143_A_G_304183827, sc00069ln687552_417242_G_A_70966666, sc00012ln1449677_470064_T_C_20535929, sc00505ln218075_188822_C_A_231896501, and sc00222ln388011_3381_C_T_149591563), one from each genomic regions were included in a stepwise regression and explained 21.8% of the FI variance (R^2^) for the Middle American subpopulation. Likewise, six markers, (sc00022ln1003704_109623_C_T_32611495, sc00265ln348200_251662_T_C_165499786, sc00394ln266395_223173_T_C_204976825, sc00615ln187153_48449_C_A_254174921, sc00074ln679062_300164_A_G_74264487 and sc01428ln78433_61756_G_A_352114770), one from each of the genomic regions were included in a stepwise regression and explained 30.5% of the FI variance (R^2^) for the Andean subpopulation. In all, 14 and 23 significant (p<0.001) marker trait associations were found for the Middle American and Andean subpopulations, respectively (Tables 2 and 3).

The research reported by Wen [26] discovered genetic factors in common bean that controlled the response to SCN Hg types 2.5.7 and 1.3.3.5.6.7 but we do not know if the accessions used by Wen were the same as in this current study. Our research used the core collection to discover genetic factors associated with Hg type 0 and divided the core collection in two major gene pools. Recent research is clearly showing that often the same phenotypes are controlled by different factors in the two gene pools [37, 38, 39] and each gene pool must be studied independently to discover factors specific to each gene pool. Therefore, it is difficult to reconcile the Wen [26] results with those we report here. However, the finding by Wen [26] 2017) of SNPs on chromosome Pv01 associated with SCN resistance does corroborate our results of SCN Hg type 0 resistance on Pv01 in the Andean gene pool as one significant SNP was found on that chromosome. In our study, a total of 2 significant genomic regions in the Middle American gene pool and 4 significant genomic regions in the Andean gene pool were found associated with resistance to SCN HG type 0 in the USDA common bean core collection. The two gene pools are estimated to diverge ~ 111,000 years ago from a common ancestor and are distinct from each other due to domestication bottlenecks and selection effects in each gene pool [29, 40]. Therefore, it is not surprising to see different genetic controls for SCN resistance in these two gene pools. The detection of multiple peaks indicated the presence of quantitative resistance which can be very useful for developing cultivars with durable resistance to SCN. Stacking or pyramiding of multiple resistance genes in the same genotype could provide durable resistance. A combination of distinct resistance genes from Andean and Mesoamerican gene pools was proposed as a way of achieving durable resistance against anthracnose and other important bean pathogens [41]. Further analysis of these genomic regions with the help of available common bean genome sequence information will be able to provide a clue about their genomic organization in both the genepools and shed light on candidate genes involved in the resistance mechanism. Also the markers identified in this study could be a useful resource for developing markers for marker-assisted selection and dissect the complexity of SCN-common bean interactions.

### Candidate gene selection

Multiple GWAS signals were colocalized with SCN resistance in dry bean. These regions contain numerous gene models and to narrow down the number of candidate gens, synteny based analysis was performed. Ample knowledge about SCN resistance genes in soybean is available but limited work has been conducted to survey the genetic basis of SCN resistance across the common bean germplasm. Comparative mapping between these two closely related species can provide the opportunity to exploit synteny among the two genomes to find resistance genes. Since SCN resistance loci in soybean are already known, we focused the search for candidate genes in common bean genomic regions based on previous information available in soybean. In soybean, linkage mapping and association analysis studies enabled the identification of *rhg1* on chromosome 18 in Peking, PI88788, PI209332, and PI437654, while a second locus, *Rhg4* on chromosome 8, was found in Peking, PI209332, and PI437654 [11, 12, 36]. Fine mapping of the *rhg1* locus on chromosome 18 confirmed that the copy number variation of a genomic segment spanning three genes (Glyma.18g02580, Glyma.18g02590 and Glyma.18g02610 within a 31 kb rhg1-b region of PI88788) was required to confer resistance [11]. These three genes (Glyma.18g02580, Glyma.18g02590 and Glyma.18g02610) code for an amino acid transporter, an alpha-soluble NSF attachment protein (SNAP) and a gamma bisabolene synthase 1-related protein, respectively. Blast analysis of these three protein sequences in the rhg1 cluster was conducted against the *P*. *vulgaris* genome (https://phytozome.jgi.doe.gov). Surprisingly, we found two *rhg1* like clusters in the *P*. *vulgaris* genome. One gene cluster containing two predicted amino acid transporters (Phvul.008G219100 and Phvul.008G219200), SNAP protein (Phvul.008G219300), and a gamma bisabolene synthase 1-related protein (Phvul.008G219400) is located on chromosome Pv08 of common bean. This gene cluster resides within the genomic region on chromosome 8 identified through association mapping analysis for the Andean subpopulation. Previous studies also showed that some parts of chromosome 18 of soybean were syntenic to chromosome Pv08 of common bean [42]. Another gene cluster, containing one amino acid transporter (Phvul.001G248000), a-SNAP protein (Phvul.001G247900), and a protein predicting gamma bisabolene synthase 1-related protein (Phvul.001G247800) was located on Pv01 of common bean. This gene cluster is present in close proximity to one significant markers on Pv01 in the Andean subpopulation identified through association mapping in our study. Comparative mapping analysis indicated that the genomic region surrounding this cluster on Pv01 is also syntenic to region surrounding rhg1 on chromosome 18 in *G*. *max* (S2 Fig). It is likely these *rhg1* like gene clusters evolved separately following the separation of the Middle American and Andean gene pools and could be involved in conferring SCN resistance in common bean because they have the same set of genes found in the soybean *rhg1* locus.

The presence of four significant marker trait association on chromosome Pv09 in Andean subpopulation also suggests another quantitative genetic control of SCN resistance in common bean. The presence of a gamma-soluble NSF attachment protein coding gene (*Phvul*.*009G218500*) near this region on chromosome 9 close to associated SNP (sc00011ln1496550_1068436_G_A_19637751) indicated an important role of SNAP proteins in SCN resistance. The same SNAP gene was upregulated in the SCN resistant common bean accession PI 533561 after 8 days of SCN infection in comparison to the non-infected PI line, but no change in expression in the susceptible GTS900 line was observed [43]. A significant peak is present on Pv07 in both the subpopulations, but the GWAS signal spans >15 Mb and there will be a large number of genes present in this region. Wen [26] also identified a significant marker trait association for Hg type 1.2.3.5.6.7 resistance on Pv07. Therefore, further work is required to narrow down this novel loci. Another loci conferring SCN resistance in soybean, *Rhg4*, is a serine hydrox methyl transferase gene (*GmSHMT08*) that is located on chromosome 8 [12]. Three SHMT genes that are homologous to *GmSHMT08* are present on Pv03, Pv06 and Pv09 of common bean. A significant marker on Pv06 was found to be associated with SCN resistance in the Middle American subpopulation in this study. Gene *Phvul*.*006G216000* (Chr06:30863765..30868299) which codes for SHMT on Pv06 is in close proximity to significant markers. A common region associated with SCN resistance in both subpopulations was found on Pv11 in dry bean. A cluster of plant resistance (R) genes controlling resistance to different pathogens such as anthracnose (Co-2), powdery mildew resistance loci (Pm1), halo blight (Rpsar-1) and bean rust (Ur-3 and Ur-11) has been found on Pv11 in common bean [44, 45]. R genes based defense response involves recognition of specific pathogen effectors by R proteins and identification of markers in close proximity suggests that R genes can also be involved in resistance to SCN.

### Sequence analysis of SNAP proteins

**Fig 4:**
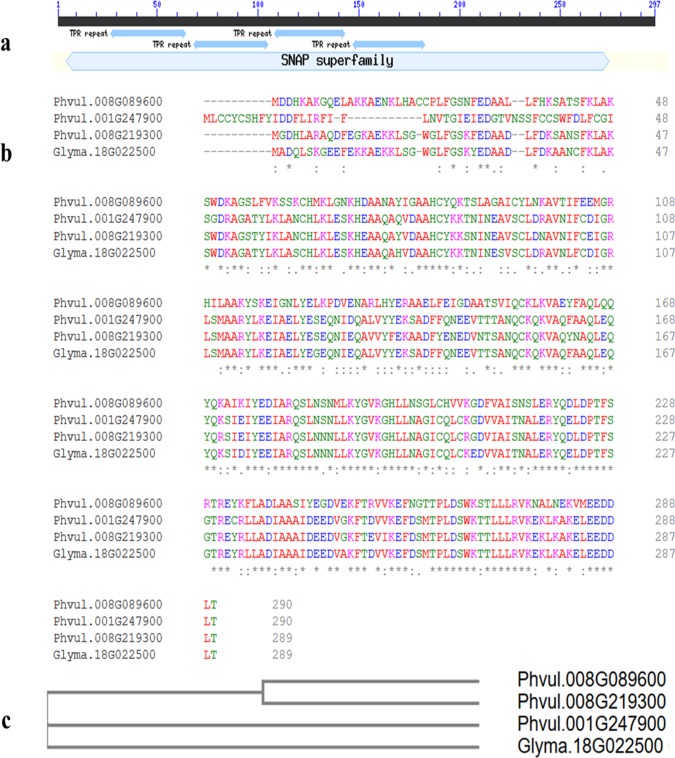
Sequence analysis of α-SNAP proteins. **(a)** Conserved residues analysis reveals that α-SNAP proteins constitute a family of proteins with a common modular architecture containing four tetratricopeptide repeat (TPR) motifs, **(b)** Comparative sequence analysis of α-SNAP proteins from *Phaseolus vulgaris* and α-SNAP protein from *Glycine max***. (c)** The alignment shows a high similarity among all four SNAPs.

The SNAP proteins are a ubiquitous member of the soluble NSF attachment protein receptor (SNARE) complex and are predicted to sustain cellular vesicle trafficking by participating in disassembly of SNARE membrane trafficking complexes [46, 47]. The SNAP proteins are found in both plants and animals. These SNAP proteins have four characteristic domains called tetratricopeptide repeat (TPR) domain consisting of 34 amino acids basic repeats and were reported to be involved in the cell cycle in yeast [48, 49]. Resistant soybean varieties contain a dysfunctional variant of the α-SNAP in the *rhg1* locus that interact poorly with NSF resulting in disruption of vesicle trafficking [50]. The virulent nematode populations of SCN produce multiple forms of bacterial-like protein containing a putative SNARE domain (*HgSLP-1*) that interact with rhg1 α-SNAP SCN resistance protein during infestation [51]. Resistance-type α-SNAPs specifically hyperaccumulate relative to susceptible wild-type α-SNAPs at the nematode feeding site and promote the death of nematodes [50]. Four SNAP genes (*GmSNAP18*, *GmSNAP11*, *GmSNAP14* and *GmSNAP02*) are found in soybean [52]. In soybean, *GmSNAP18* (Glyma.18G022500) is required for *rhg1* type SCN resistance. Furthermore, in soybean *rhg1*, a Peking-type *GmSNAP18* is sufficient for resistance to SCN in combination with *Rhg4* [53]. A high amount of conserved gene sequences were observed in *GmSNAP18*/*GmSNAP11* and *GmSNAP14*/*GmSNAP02* indicating the results of duplication and also indicating that actually two SNAP proteins contribute to SCN resistance [52]. Based on a homology search of SNAP proteins, three α-SNAPs (two on Pv08 and one on Pv01) were identified in common bean genome. Sequence alignment analysis of all three *P*. *vulgaris* α-SNAP proteins (Phvul.008G219300, Phvul.008G089600, Phvul.001G247900) found in the common bean and soybean α-SNAP (Glyma.18G022500) demonstrated a high similarity (Fig 4). Protein sequence of Phvul.008G219300 was highly similar to Phvul.001G247900, Glyma.18G022500 and Phvul.008G089600 with 88% (2e-164), 86% (2e-156) and 61% (7e-132) similarity, respectively. Protein sequence of Phvul.001G247900 was highly similar to Glyma.18G022500 with 94% (4e-171) similarity but show only 59% similarity with Phvul.008G089600 (4e-106). SNAP genes coded by Phvul.008G219300 and Phvul.001G247900 are part of two *rhg1* like gene clusters in common bean, however, the SNAP gene coded by Phvul.008G089600 did not have two other genes of the *rhg1* cluster close by. Bayless et al. [50] hypothesized that higher expression of *rhg1* resistance type α-SNAPs impair NSF function and disrupt vesicle trafficking, thereby dissolving syncytia, a nematode feeding site. However, these deleterious effects are tolerated due to presence of wild type α-SNAPs in nearby cells. Further work will be required to determine if these gene have any role in SCN resistance or whether they are wild type α-SNAP in common bean to counteract cytotoxicity.

## Conclusion

SCN has the potential to cause severe economic impacts in all common bean production regions of the USA. Thus, the identification of common bean genomic regions of resistance/susceptibility that interact with SCN effectors is important to the eventual deployment of durable genetic resistance against this adaptable pest. The US soybean breeding community has been successful in its efforts to identify SCN resistant genotypes; however, the genetic basis of resistance is very limited. Similar assumptions could be made for common bean as well because of the evolutionary relationships. This is the first study to report use of GWAS and comparative mapping to decipher genomic regions associated with SCN HG 0 resistance reactions of common bean in the two core collection gene pools. A gene cluster which codes for the *rhg1* type of resistance in soybean was found in both the common bean genepools. Sequences show high similarity with the soybean *rhg1* locus. The results provide foundational genetic information to select the genotypes and candidate genes for SCN resistance in the core collection and can be a source for the effective deployment of SCN resistance in elite common bean lines.

## Supporting information

S1 Fig Linkage disequilibrium maps for chromosome 8. Single nucleotide polymorphisms associated with soybean cyst nematode resistance identified by association mapping (*p*-value < 0.001) are indicated by black arrows. The shades of red colors on the diagonal show high squared correlation coefficients between the pair of markers.
(TIF)

S2 Fig Synteny analysis of genomic region containg *rhg1* like gene cluster in common bean with soybean. Visualization of synteny maps of a1.43Mb region covering gene clusters similar to *rhg 1* and *rhg1* (in black box) in *Phaseolus vulgaris* (Pv08 and Pv01) and *Glycine max* (Gm18), respectively.
(TIF)

S1 Table List of SCN resistant accessions. Accessions having female index less than 10 were considered resistant.
(XLSX)
