# Supplementary figures and images for: Genome wide association study discovers genomic regions involved in resistance to soybean cyst nematode (Heterodera glycines) in common bean
Source: PLoS One. 2019 Feb 7;14(2):e0212140. doi: 10.1371/journal.pone.0212140 (PMC6366866; doi:10.1371/journal.pone.0212140)

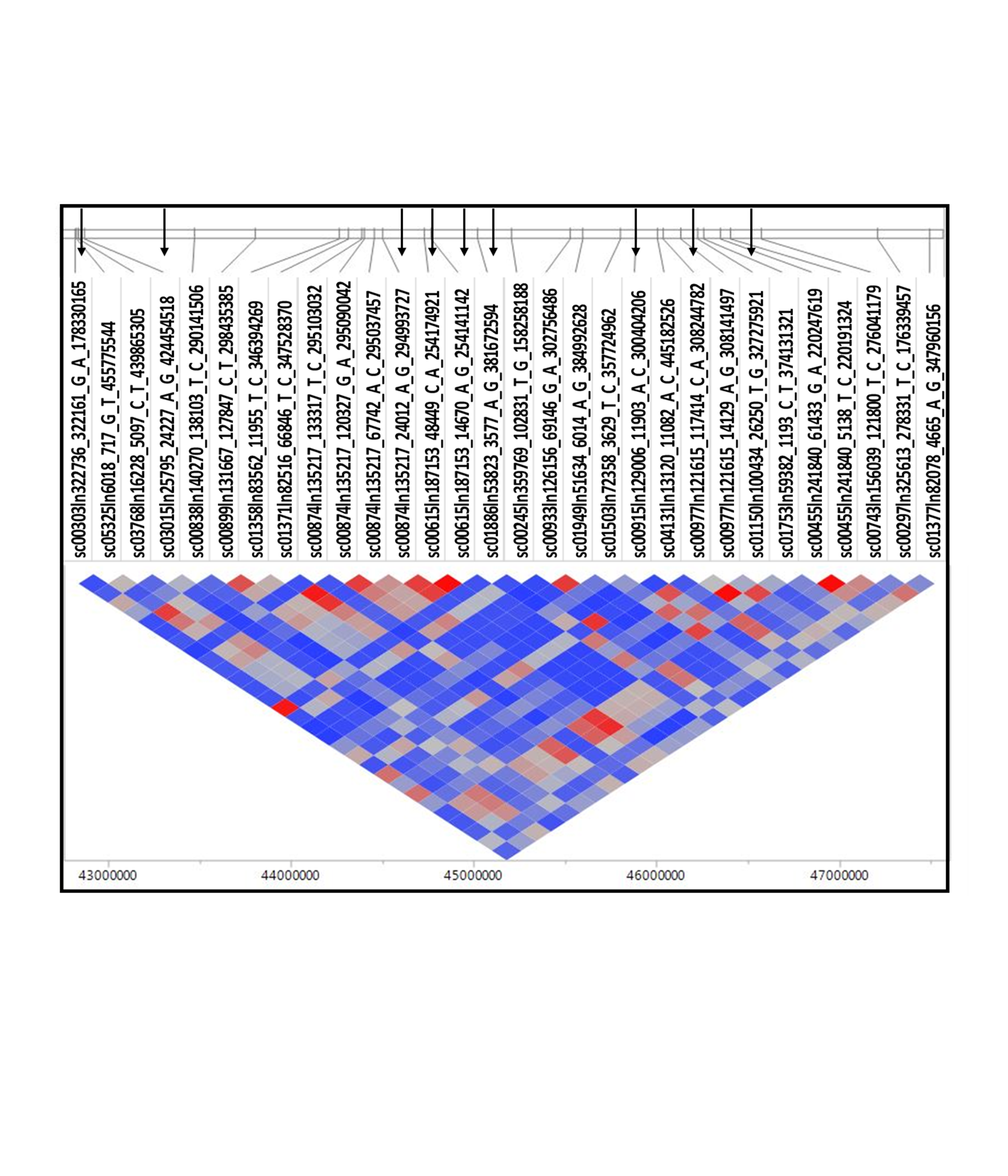

Supplement: S1 Fig — Single nucleotide polymorphisms associated with soybean cyst nematode resistance identified by association mapping (p-value < 0.001) are indicated by black arrows. The shades of red colors on the diagonal show high squared correlation coefficients between the pair of markers. (TIF) [file pone.0212140.s001.tif]

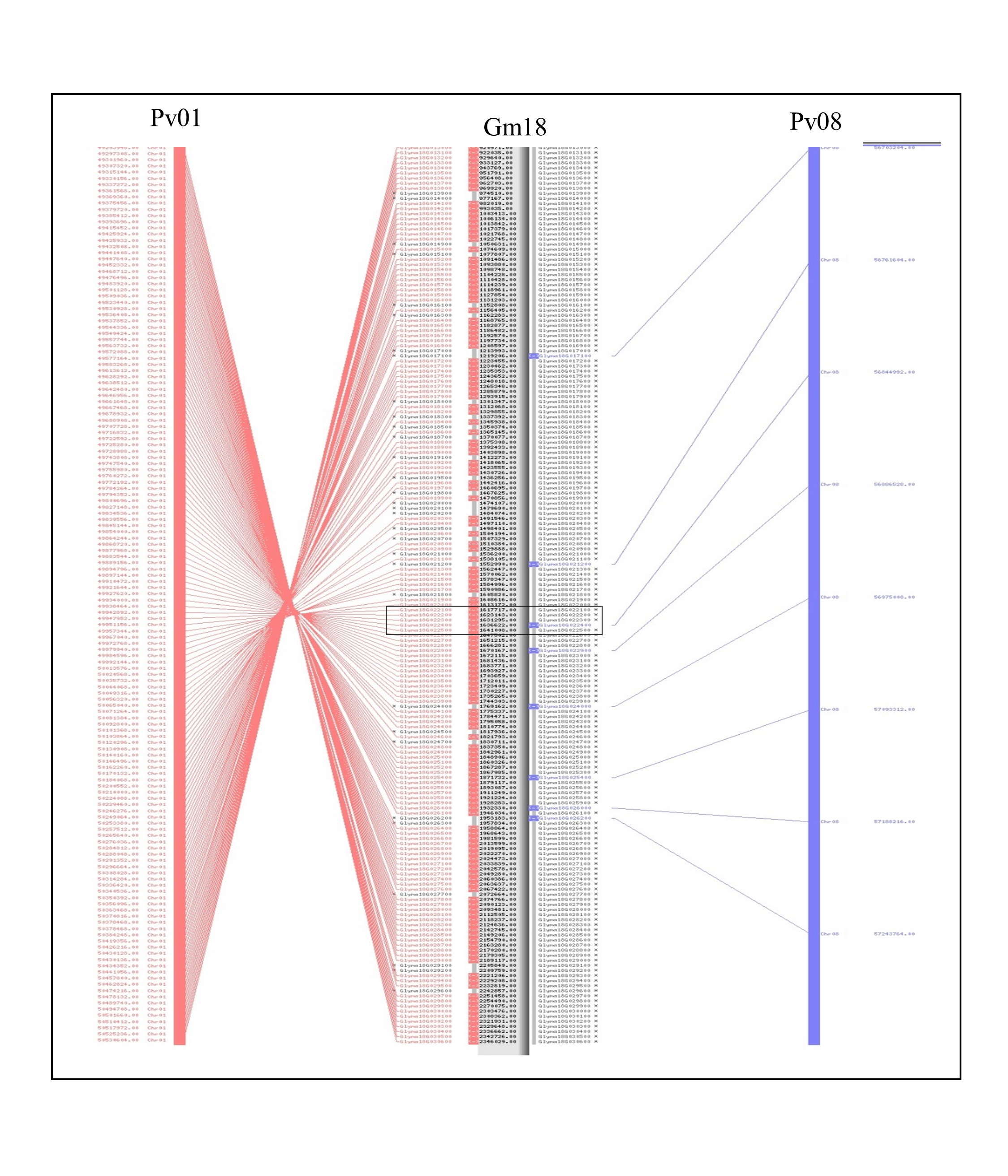

Supplement: S2 Fig — Visualization of synteny maps of a1.43Mb region covering gene clusters similar to rhg 1 and rhg1 (in black box) in Phaseolus vulgaris (Pv08 and Pv01) and Glycine max (Gm18), respectively. (TIF) [file pone.0212140.s002.tif]
